# Association Study between Cervical Lesions and Single or Multiple Vaccine-Target and Non-Vaccine Target Human Papillomavirus (HPV) Types in Women from Northeastern Brazil

**DOI:** 10.1371/journal.pone.0132570

**Published:** 2015-07-15

**Authors:** Bárbara Simas Chagas, Manola Comar, Ana Pavla Almeida Diniz Gurgel, Sérgio Paiva, Silva Seraceni, Antonio Carlos de Freitas, Sergio Crovella

**Affiliations:** 1 Laboratory of Molecular Studies and Experimental Therapy, Department of Genetics, Federal University of Pernambuco, Pernambuco, Brazil; 2 Institute for Maternal and Child Health - IRCCS “Burlo Garofolo” - Trieste, Italy; 3 University of Trieste, Trieste, Italy; Istituto Nazionale Tumori, ITALY

## Abstract

We performed an association between high-grade squamous intraepithelial lesions (HSIL), low-grade squamous intraepithelial lesions (LSIL) and single or multiple vaccine-target as well as non-vaccine target Human papillomavirus (HPV) types. Using bead-based HPV genotyping, 594 gynecological samples were genotyped. An association between squamous intraepithelial lesion (SIL) and presence of HPV16, 18, 31, 58 and 56 types were calculated. The risk was estimated by using odds ratio (OR) and 95% of confidence intervals (CI). A total of 370 (62.3%) women were HPV positive. Among these, 157 (42.7%) presented a single HPV infection, and 212 (57.3%) were infected by more than one HPV type. HPV31 was the most prevalent genotype, regardless single and multiple HPV infections. Single infection with HPV31 was associated with LSIL (OR=2.32; 95%CI: 1.01 to 5.32; *p*=0.04); HPV31 was also associated with LSIL (OR=3.28; 95%CI: 1.74 to 6.19; *p*= 0.0002) and HSIL (OR=3.82; 95%CI: 2.10 to 6.97; *p*<0.001) in multiple HPV infections. Risk to harbor cervical lesions was observed in multiple HPV infections with regard to the HPV56 (OR=5.39; 95%CI: 2.44 to 11.90; *p*<0.001for LSIL; OR=5.37; 95%CI: 2.71 to 10.69; *p*<0.001) and HPV58 (OR=3.29; 95%CI: 1.34 to 8.09; *p*=0.0091 for LSIL; OR=3.55; 95%CI: 1.56 to 8.11; *p*=0.0026) genotypes. In addition, women coinfected with HPV16/31/56 types had 6 and 5-fold increased risk of HSIL (OR=6.46; 95%CI: 1.89 to 22.09; *p*=0.002) and LSIL (OR=5.22; 95%CI: 1.10 to 24.70; *p*=0.03), respectively. Multiple HPV infections without HPV16/18 has 2-fold increased risk of HSIL (OR=2.57; 95%CI: 1.41 to 4.70; *p*=0.002) and LSIL OR=2.03; 95%CI: 1.08 to 3.79; *p*=0.02). The results of this study suggest that single and multiple vaccine target as well as non-vaccine target HPV types are associated with LSIL and HSIL. These finding should be taken into consideration in the design of HPV vaccination strategies.

## Introduction

Clinical and epidemiological studies report that cervical infection with High Risk Human papillomavirus (HR HPV) is necessary but not sufficient to cause the development of cervical cancer [1,2]. Additional risk factors are likely to be involved in the development of cervical cancer, including multiple HPV infections [3].

Persistent infection with HR HPV is considered as the main cause of cervical lesions and cervical cancer [4]. To date, 184 HPV types have been identified (http://www.hpvcenter.se/html/refclones.html) and approximately 40 of them are known to be able to infect mucosal areas including the epithelium of the ano-genital tract. The classification of HPV types is based on the intrinsic oncogenic potential of these viruses, where 15 oncogenic genotypes are associated with the majority of cervical lesions and cervical cancer [5]. Among these, epidemiological data showed that vaccine-target HPV (VT HPV) 16 (Ahpa-9) and 18 (Alpha-7) types are responsible for 70% of the cases of invasive cervical cancer worldwide [6]. The remainder 30% of cervical cancer rates are caused by other genotype target of 9-Valent HPV vaccine (HPV31, 33, 45, 52 and 58) as well as non-vaccine HPV types (NV HPV) (such as HPV26, 35, 39, 53, 56, 66, 68, 73) [7].

Multiple HPV infections are commonly found in molecular epidemiologic studies [8–20]. However, the clinical importance of these multiple HPV types in the modulation of the risk to squamous intraepithelial lesions (SIL) remains controversial. Some studies have shown an association between low squamous intraepithelial lesion (LSIL) or high squamous intraepithelial lesions (HSIL) in women infected with multiple HPV types [11–13,16,21]. In addition, studies have demonstrated that multiple HPV types occur significantly more frequently than predicted by chance [12,13,16]. In this scenario, some studies have demonstrated that specific combination of HPV types, including the genotypes target of 9-Valent vaccine and NV HPV, may act synergistically to induce any SIL [13,16].

Despite some evidences reported cross-protection of HPV16/18 vaccine among Alpha-9 (HPV31, 33, 52) and Alpha-7 (HPV45) species [22–30], there are poor indications whether this vaccine indirectly increases or decreases the prevalence and risk to develop cervical lesions caused by these HPV types [16,31,32]. Hence, studies concerning the prevalence and risk that a women have to harbor cervical lesions when infected with single or multiple VT HPV as well as NV HPV are important to devise new vaccine strategies tackling viruses. In this study, we reported the risk of cervical lesions (LSIL and HSIL) in the presence of single and multiple VT HPV as well as NV HPV genotypes in a group of HPV infected women from Northeastern Brazil.

## Methods

### Study group

The samples evaluated in this study were obtained by cervical scraping from 594 patients who volunteered to take part in cervical cancer screening at the Gynecological Clinic at the Clinical Hospital (n = 293) and Oswaldo Cruz University Hospital (n = 130) at Recife, Pernambuco State, Northeastern Brazil, and at the Center for Integral Attention to Women’s Health (n = 171) at Aracaju, Sergipe State, Northeastern Brazil. The study included 91 women (mean age 31.8 ± 10.1; median 29) with low-grade squamous intraepithelial lesions (LSIL), 113 women (mean age 37.5 ± 12.9; median 34) with high-grade squamous intraepithelial lesions (HSIL) and 390 women (mean age 34.8 ± 10.1; median 34) with normal cytological results. On the other hand, the exclusion criteria were current pregnancy, undergone hysterectomy or being HIV-positive.

Women attended at the three screening centers (two in Pernambuco, one in Sergipe) were resident in the Metropolitan area of Recife and Aracaju and nobody came from rural areas due to the travel expenses; the costs of patients’ dislocation, is an important social issue to be considered when dealing with individuals enrolled in public health structures; in fact, as it is already known in North East Brazil, people using public health service are from the low income portion of the population with little or no facilities to travel around, being the health service predominant in medium upper social classes. So, patients had very similar socio-economical background, living all in the metropolitan areas of Recife and Aracaju, characterized by low-income economic status, associated with poor scholar training and unemployment (or temporal employment with minimal salary).

All women participating in this study had not been vaccinated against HPV. The cervical cells collected with cytobrush were placed in phosphate-buffered saline (PBS) pH 7.4 and stored at -80°C prior to DNA extraction.

The samples of cervical cells were processed for DNA extraction using the DNeasy Blood and Tissue Kit (Qiagen), in accordance with the following steps: suspension of the cell pellet in PBS (pH 7.4), cell lysis, purification, washing and drying of the material to obtain the DNA elution.

### Ethics Statement

All clinical investigation developed in this study was conducted according to the principles expressed in the Declaration of Helsinki. The patients were enrolled in the period between February 2011 and June 2013, and were informed about the objectives of the research. We obtained approval of the Ethical Committee (Research Ethics Committee in Human Beings—Hospital Complex Oswaldo Cruz—HUOC/PROCAPE 64/2010; Research Ethics Committee—Health Sciences Center/Federal University of Pernambuco—CEP/CCS/UFPE N° 491/11) and all women signed the consent. The inclusion criteria were to agree in participating to the study and be at least 18 years.

### HPV type-specific E7 PCR bead-based multiplex genotyping (BioPlex)

The multiplex HPV type-specific E7 PCR utilizes HPV type-specific primers targeting the E7 region for the detection of several HR-/pHR-HPV types and 2 LR-HPV types, with detection limits ranging from 10 to 1.000 copies of the viral genome. The amplicon size varies between 210 and 258 bp. Two primers for amplification of the beta-globin gene were also included to provide a positive control for the quality of the template DNA [20]. Each reaction mixture was analysed by Multiplex Human Papillomavirus Genotyping (MPG) using the Luminex technology (Luminex Corporation, Austin, TX) as described previously [32,33].

### Statistical analysis

The most prevalent HPV 16, 18, 31, 56 and 58 types were analyzed separately. The odds ratio of LSIL and HSIL, with single or multiple HPV infections were calculated in comparison with normal group. Odds ratios (OR) and 95% confidence intervals (CI) are reported through the text and in the Tables. Statistical analyses were performed by using MediCal version 14.10.2 http://www.medcalc.org/index.php. To evaluate the influence of cofactors (age groups, number of sexual partners and oral contraceptive use) in HPV infected patients LSIL and HSIL we used the logistic regression model. The comparison between the groups LSIL, HSIL and Normal cytology was made by the chi-square test using the statistical software R (R Core Team, 2015). Significance was defined as P<0.05 for all tests.

## Results

A total of 594 cytological samples classified as normal cytology, HSIL and LSIL were genotyped by using BioPlex platform. The prevalence of HPV was 62.3% (370/594), where 42.7% (158/370) of the samples had a single HPV infection, and 57.3% (212/370) were infected with multiple HPV types (Table A in S1 File). HPV negative results were detected in 224 (37.7%) samples, and the human control β-globin was positive in all samples, excluding false negative findings.

Age groups, number of sexual partners and eventual use of contraceptive of studied women classified on the basis of cervical lesions severity are summarized in Table 1. Statistical analysis among group of women with normal cytology, LSIL and HSIL corrected according to the co-factors age, number of sexual partners, use of oral contraceptive and HPV infection was performed to show possible differences with expected values, due to the influence of the variables mentioned above.

**Table 1:** Characteristics of women according to the severity of cervical lesions.

		Normal cytology group		LSIL group		HSIL group	
Characteristics	Total no. of patients	No. of patients	No. of patients with HPV (%)	No. of patients	No. of patients with HPV (%)	No. of patients	No. of patients with HPV (%)
Age group (years)							
<30	217	145	91 (62.75)	52	41 (78.84)	38	34 (89.47)
30–39	187	119	68 (57.14)	17	12 (70.58)	33	26 (74.28)
40–49	133	92	48 (52.17)	16	10 (62.50)	25	16 (64.00)
50–59	44	31	8 (25.80)	5	2 (40.00)	8	8 (100)
≥60	13	3	1 (33.33)	1	1 (100)	9	7 (77,77)
Number of sexual partners							
≤2	387	287	161 (56.09)	49	35 (71.42)	51	40 (78.43)
3 a 4	144	66	32 (48.48)	19	14 (73.68)	40	31 (77.50)
≥5	63	37	23 (62.16)	23	17 (73.91)	22	20 (90.90)
Oral contraceptive use							
No	429	294	158 (73.15)	53	35 (66.03)	82	64 (78.04)
Yes	165	96	58 (26.85)	38	31 (81.57)	31	27 (87.09)

Normal cytology *P*-value: Age (<0.001), Sexual Patners (<0.001), Contraceptive use(<0.001)

LSIL *P*-value: Age (<0.001), Sexual Patners (0.002), Contraceptive use(0.115)

HSIL *P*-value: Age(<0.001), Sexual Patners (0.036), Contraceptive use (<0.001)

**Fig 1:**
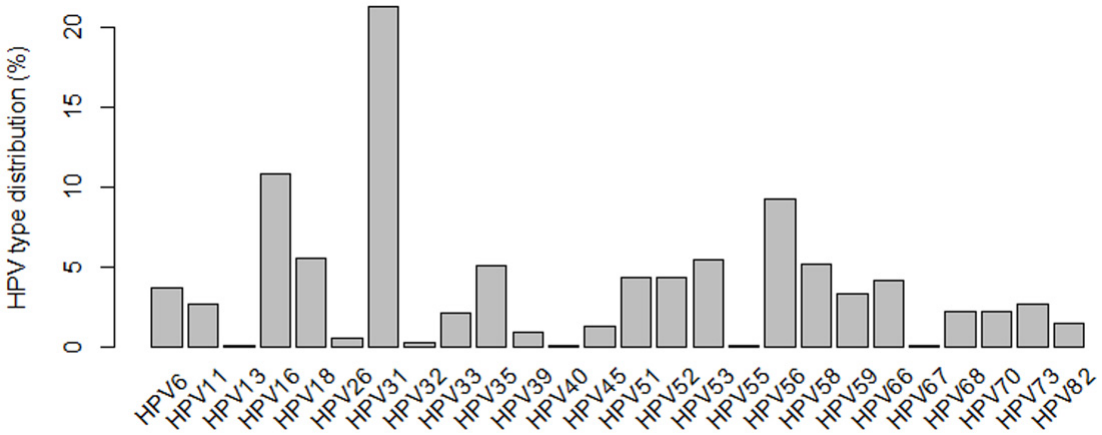
HPV diversity classified as single or multiple HPV infections found in normal and abnormal cytology of women from Northeast Brazil.

**Fig 2:**
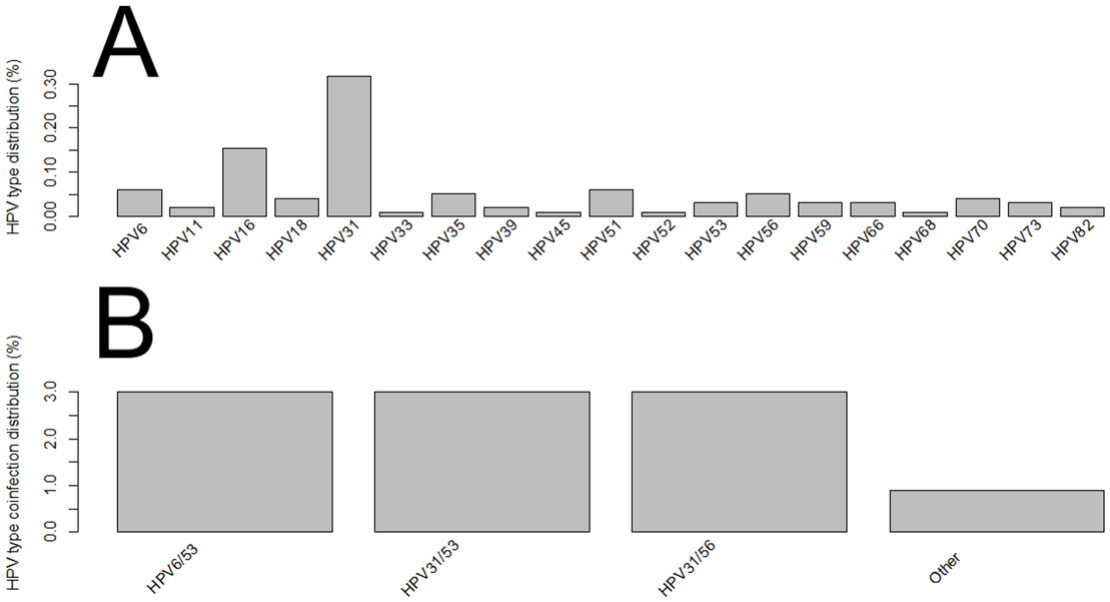
Distribution of HPV types in normal cervical samples of women from Northeast Brazil. (A) Single infection. (B) Multiple HPV infections.

**Fig 3:**
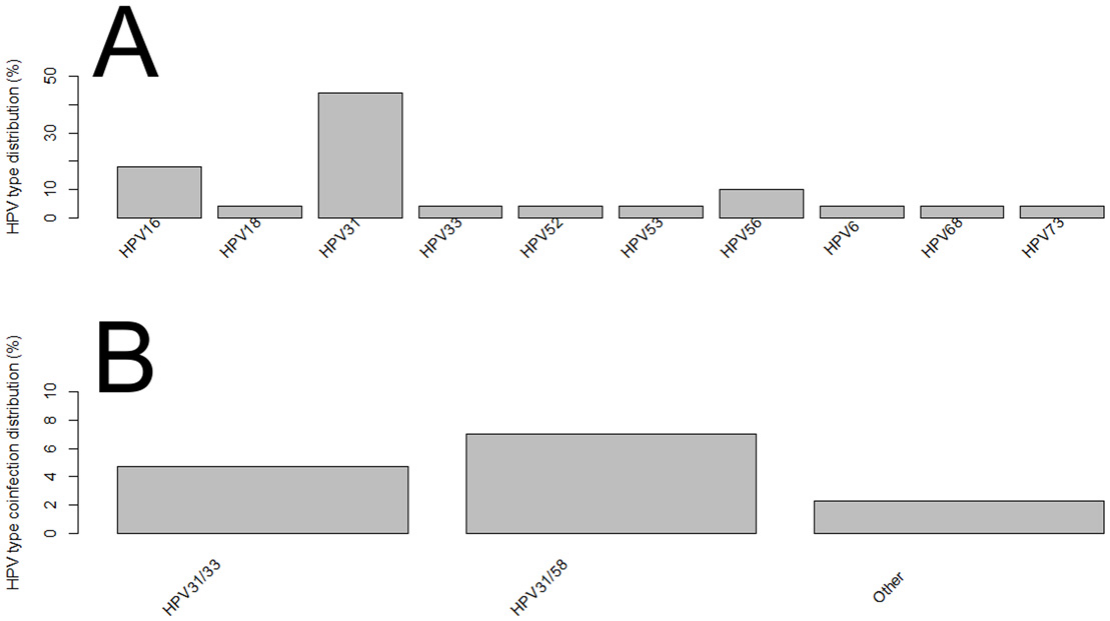
Distribution of HPV types in LSIL cervical samples of women from Northeast Brazil. (A) Single infection. (B) Multiple HPV infections. *Other*: HPV types which have a frequency below two.

**Fig 4:**
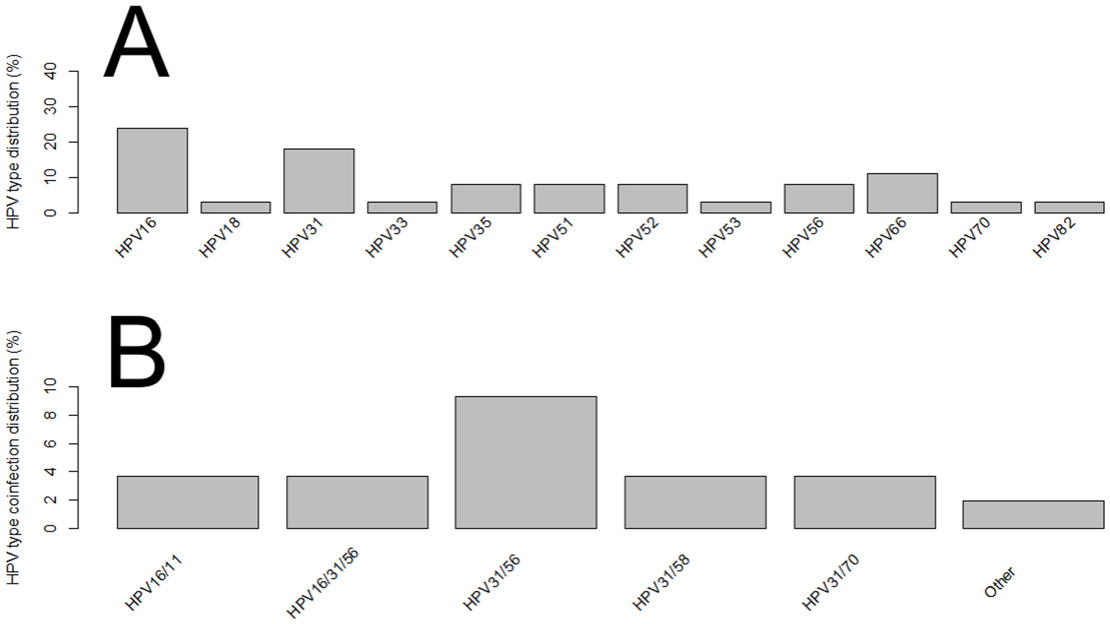
Distribution of HPV types in HSIL cervical samples of women from Northeast Brazil. (A) Single infection. (B) Multiple HPV infections. *Other*: HPV types which have a frequency below two.

The most commonly detected genotypes were HPV31 (13% in single infections; 30% in multiple infections), HPV16 (7.6% in single infections; 14.3% in multiple HPV infections), and HPV56 (2.7% in single infections; and 16% in multiple infections) (Fig 1 and Table A in S1 File). We also stratified the cervical samples according to the cytological findings and HPV type distribution (Table B in S1 File). A total of 57.6% (213/370) of women had normal cytology, in which HPV31, HPV16, HPV35 were the three most frequent types in single infections, whereas HPV6/53, HPV31/53 and HPV31/56 types were most frequent in co-infections (Fig 2A and 2B). HPV31, HPV16 and HPV56 types were the three most frequent genotypes found in LSIL with single HPV infections, while HPV31/58 and HPV31/33 were the most prevalent types found in LSIL with co-infections (Fig 3A and 3B). Regarding women diagnosed with HSIL, HPV16, HPV31, HPV66, HPV35, HPV51, HPV52 and HPV56 were most commonly detected in single HPV infection, while HPV31/56 was the most frequent co-infections observed in this group (Fig 4A and 4B).

Single infection with HPV16 has 4.5-fold increased risk of HSIL (OR = 4.5; 95%CI: 1.81 to 11.3; *p* = 0.0012), although this was not observed in women with LSIL (*p* = 0.25) (Table 2). Moreover, single infection with HPV16 in HSIL was associated with the number of sexual partners (3–4 partners) (OR = 23.7; 95%CI: 2.20 to 697.20; *p* = 0.0212) (Table 3). With regard to multiple HPV infections, HPV16 was associated with LSIL (OR = 5.5; 95%CI: 2.48 to 12.18; *p*<0.001) and HSIL (OR = 5.1; 95%CI: 2.48 to 10.31; *p* **<**0.001) (Table 2). Despite HPV18 as single HPV infection was not associated with LSIL (*p* = 0.62) and HSIL (*p* = 0.07), an association was observed between the presence of HPV18 in multiple HPV infections and HSIL (OR = 2.58; 95%CI: 1.05 to 6.36; *p* **=** 0.03) (Table 2). Single infection with HPV31 was associated with LSIL (OR = 2.32; 95%CI: 1.01 to 5.32; *p* = 0.04), but not HSIL (*p* = 0.32). However, co-infections with HPV31 showed association in both LSIL (OR = 3.28; 95%CI: 1.74 to 6.19; *p* = 0.0002) and HSIL (OR = 3.82; 95%CI: 2.10 to 6.97; *p* **<**0.001) (Table 2). In addition, co-infections with HPV31 has 4.5-fold increased risk of HSIL in women who have 3–4 partners (OR = 4.52; 95%CI: 1.43 to 14.99; *p* = 0.0108) (Table 3). An association was also observed between multiple HPV infections and cervical lesions with regard to the HPV56 (OR = 5.39; 95%CI: 2.44 to 11.90; *p*<0.001, for LSIL; OR = 5.37; 95%CI: 2.71 to 10.69; *p* **<**0.001, for HSIL) and HPV58 (OR = 3.29; 95%CI: 1.34 to 8.09; *p* = 0.0091, for LSIL; OR = 3.55; 95%CI: 1.56 to 8.11; *p* = 0.0026, for HSIL) (Table 2). Co-infections with HPV56 has 10.9-fold increased risk of HSIL in women who have 3–4 partners (OR = 10.94; 95%CI: 2.42 to 61.62; *p* = 0.0032) (Table 3).

**Table 2:** Distribution of single and multiple HPV infections in normal, LSIL and HSIL cervical samples.

	Normal	LSIL	OR (95% CI) *P-value	HSIL	OR (95% CI) *P-value
HPV16 (SI)	14	4	1.99 (0.61–6.52) 0.25	9	4.52 (1.81–11.27) 0.0012
HPV16 (MHI)	19	15	5.49 (2.48–12.18) < 0.001	19	5.05 (2.48–10.32) < 0.001
HPV18 (SI)	4	1	1.74 (0.19–16.19) 0.62	1	2.27 (0.93–5.53) 0.07
HPV18 (MHI)	21	7	2.32 (0.89–6.01) 0.08	8	2.584 (1.05–6.36) 0.03
HPV31 (SI)	30	10	2.32 (1.01–5.32) 0.046	7	1.58 (0.63–3.96) 0.32
HPV31 (MHI)	53	25	3.28 (1.74–6.19) 0.0002	33	3.82 (2.10–6.97) < 0.001
HPV56 (SI)	5	2	2.78 (0.51–15.12) 0.23	3	3.88 (0.93–16.08) 0.06
HPV56 (MHI)	22	15	5.39 (2.44–11.90) < 0.001	22	5.378 (2.71–10.69) < 0.001
HPV58 (SI)	-	-		-	
HPV58 (MHI)	19	9	3.29 (1.34–8.09) 0.0091	11	3.55 (1.56–8.11) 0.0026
HPV16 (MHI) excluding HPV31/56	7	4	3.98 (1.09–14.56) 0.03	7	5.76 (2.02–16.37) 0.001
HPV31 (MHI) excluding HPV16/56	37	14	2.63 (1.25 to 5.54) 0.01	14	2.48 (1.19–5.19) 0.01
HPV56 (MHI) excluding HPV16/31	10	4	2.78 (0.81–9.55) 0.1	4	2.78 (0.78–8.54) 0.11
HPV16/31/56 types	4	3	5.22 (1.10–24.70) 0.03	5	6.46 (1.89–22.09) 0.002
Overall HPV excluding HPV16	96	28	2.03 (1.12 to 3.68) 0.01	35	2.55 (1.43–4.55) 0.015
Overall HPV excluding HPV18	64	36	2.67 (1.51–4.71) 0.0007	46	3.20 (1.84–5.57) < 0.001
Overall HPV excluding HPV16/18	79	25	2.03 (1.08 to 3.79) 0.02	22	2.57 (1.41–4.70) 0.002

SI—Single infection. MHI- Multiple HPV infections.

(*) *P*-value and ORs in comparison with normal group.

(-) HPV DNA not detected.

**Bold**- statistically significant.

**Table 3:** Distribution of single and multiple HPV infections in normal and HSIL cervical samples associated with risk factors.

	Control	HSIL	OR (95% CI) *P-value	Risk factor
HPV31 (MHI)	76	32	4.52 (1.43–14.99) 0.0108	3–4 sexual partners
HPV56 (MHI)	36	21	10.94 (2.42–61.62) 0.0032	3–4 sexual partners
HPV16 (SI)	20	9	23.68 (2.20–697.10) 0.0212	3–4 sexual partners

SI—Single infection. MHI- Multiple HPV infections.

(*) *P*-value and ORs in comparison with normal group (Control).

**Bold**- statistically significant.

Multiple HPV infections with HPV16 and excluding HPV31/56 types were associated with LSIL (OR = 3.97; 95%CI: 1.09 to 14.56; *p* = 0.03) and HSIL (OR = 5.75; 95%CI: 2.02 to 16.37; *p* = 0.001) (Table 2). Similarly, multiple HPV infections with HPV31 and excluding HPV16/56 had a 2.5-fold increased risk to LSIL (OR = 2.63; 95%CI: 1.25 to 5.54; *p* = 0.01) and HSIL (OR = 2.48; 95%CI: 1.19 to 5.19; *p* **=** 0.01) (Table 2). In contrast, multiple infections with HPV56 and without HPV16/31 were not associated with both LSIL (*p* = 0.1) and HSIL (*p* = 0.11) (Table 2). Multiple infections with HPV16/31/56 had a 5-fold increased risk to LSIL (OR = 5.22; 95%CI: 1.10 to 24.70; *p* = 0.03) and 6-fold increased risk to HSIL (OR = 6.46; 95%CI: 1.89 to 22.09; *p* = 0.002) (Table 2). In addition, multiple HPV infections excluding all women infected with HPV16 had a 2-fold increased risk to LSIL (OR = 2.03; 95%CI: 1.12 to 3.68; *p* = 0.01) and HSIL (OR = 2.55; 95%CI: 1.43 to 4.55; *p* = 0.015) (Table 2). Similarly, multiple infections excluding HPV18 had a 2-fold increased risk to LSIL (OR = 2.67; 95%CI: 1.51 to 4.71; *p* = 0.0007) and HSIL (OR = 3.2; 95%CI: 1.84 to 5.57; *p*<0.001). Multiple HPV infections without HPV16/18 types had a 2-fold increased risk to HSIL (OR = 2.57; 95%CI: 1.41 to 4.70; *p* = 0.002) and LSIL (OR = 2.03; 95%CI: 1.08 to 3.79; *p* = 0.02) (Table 2).

## Discussion

We performed an association study between cervical lesions and single and multiple VT HPV as well as NV HPV types. Our results showed several meaningful findings, as follow: 1) HPV31 is the most prevalent genotype found in women from Northeastern Brazil; 2) NV HPV56 is the third most frequent genotype; 3) Single infections with either NV HPV51 and HPV56 are frequent in HSIL; 4) Co-infections with HPV31 and NV HPV56 are the most frequent in HSIL; 5) Only single HPV infection with either HPV16 and HPV31 is associated with any SIL; 6) Co-infections with HPV31 had a 3-fold increased risk to any SIL; 7) Co-infections with NV HPV56 have four to five times more risk to any SIL; 8) Co-infections with HPV58 have three times more risk to any SIL; 9) Co-infections HPV31 and NV HPV56 excluding HPV16 have four to five times more risk to any SIL; 10) Multiple HPV infection without HPV16/18 have two times more risk to any SIL. Taken together, these findings suggest an association between the presence of HPV types and the risk to develop any SIL in women from Northeastern Brazil.

To date, there is no consensus whether multiple HPV types occur randomly or through competitive or cooperative relationship. Studies have demonstrated that infection with multiple HPV types occurs more frequently than predicted by chance [12,13,16]. In this perspective, some of these studies have suggested that multiple HPV types can act synergistically in cervical carcinogenesis [12,13,16]. For instance, Trottier et al. demonstrated that HPV51, 52, 56, and 58 types might cooperate with HPV16 to produce any SIL or cancer [21]. Dickson et al. showed that HPV52, 53, 81, and 83 were more likely to occur in multiple HPV infections, while HPV16, 58, and 66 seem to be less likely to occur in multiple HPV infections with other types [12]. Furthermore, HPV51 and HPV58 may develop carcinogenesis more rapidly and more extensively when infected with other HPV types [21]. Moreover, viral ecological data have shown that NV HPV72 and 81 types have a positive relationship, while HPV33 and 66 have a negative relationship [12]. In addition, Munagala et al. [2009] showed that radiotherapy treatment for cancer failed five times more in women infected with multiple NV HPV infections when compared to women with an unique infection [34]. In the current analysis, co-infections with either HPV16 or HPV18 showed the highest risk to harbour cervical lesions when compared to single infection. Similarly, multiple HPV types also were associated with any SIL. Single or multiple HPV infections with HPV31 have 2 to 3-fold increased risk for any SIL. Similarly, co-infections with HPV56 and HPV58 were also more likely to have any SIL when compared to single infection. These findings are consistent with other studies, where women with multiple HPV infections showed increased risk of both cervical intraepithelial neoplasia grade 2 (CIN2) and HSIL when compared with single infection [9,12,13,16,17,21,35,36]. Even when we excluded women with HPV16/18 from the analysis, multiple HPV types have 2-fold increased risk for both LSIL and HSIL. Simultaneously, in multivariable logistic regression models, evaluating the exposure to an important risk factor for HPV infection such as sexual behaviors, our results suggested that single HPV infection with HPV16 have 23.6-fold increased risk for HSIL when associated with the number of sexual partners (3–4 partners). Similarly, when associated with the number of sexual partners (3–4 partners) our results suggested that co-infections with HPV31 and HPV56 have 4.5-fold and 10.9-fold increased risk for HSIL, respectively. The associations with age and contraceptive use were not observed in our study.

Several authors have shown that HPV16 is the most prevalent in Brazil [19,20,37–45]. In contrast, the current study showed HPV31 as the most prevalent genotype found in Northeastern Brazil; however we must considering that we have analyzed women living only in metropolitan areas of Recife and Aracaju, capital cities, of just two states, Pernambuco and Sergipe, of North East Brazil. The high prevalence of HPV31 in Northeastern Brazil was also reported in our previous studies, always conducted on women from metropolitan areas [20,37,46,47], in which HPV 16 was the most prevalent genotype followed by HPV31 type. We speculate that high incidence of HPV31 circulating in the Northeast region of Brazil could be related to the high frequency of some HPV31 variants. Several studies demonstrated that lineage variants of HPVs are associated to infection persistence and progression of the cervical lesion and cervical cancer [48–66]. In this context, studies performed by Chagas et al. [46,47] and Gurgel et al. [67] show that the A and C lineage variants of HPV31 are the most frequent in women from Northeast region of Brazil. In addition, epidemiological studies also have demonstrated that A and C lineage variants of HPV31 are the most oncogenic when compared to the B variant. Hence, epidemiological studies with regard to the HPV31 variants circulating should be performed to elucidate whether A/C variants of HPV31 are responsible for high incidence of this HPV type in Northeastern Brazil.

For the first time, HPV56 was observed as the third most common HPV (9.24%) found in Northeastern Brazil. The discrepancy between the HPV prevalence studies may be attributed to different HPV DNA detection strategies. In this study, we used a multiplex HPV genotyping, which is a simple bead-based high-throughput hybridization method that allows to detect several HR HPV [68]. In addition, multiplex HPV genotyping used in the current study appears to be more sensitive when compared to MY09/11 and GP05+/06+ consensus primers, Hybrid Capture II assay and Linear Array HPV genotyping test [33,48].

Several evidences have reported cross-protection of the bivalent vaccine (HPV16/18; Cervarix, GlaxoSmithKline Biologicals) and the quadrivalent vaccine (HPV/6/11/16/18; Gardasil, Merck) against HPV31, 33 and 45 types [22–30]. However, there is little evidence whether this vaccine indirectly has an impact in the increase or decrease of some HPV types [16,30,31]. For instance, Kahn et al. demonstrated that prevalence of NV HPV types increases significantly among vaccinated women but not among unvaccinated women [30]. Hence, studies concerning the prevalence and risk of NV HPV are important to devise new vaccine strategies focusing these viruses. Recently, bivalent and quadrivalent HPV vaccines have been released in Brazil for application in females aged from 9 to 26 years old, and currently quadrivalent vaccine that immunizes against HPV types 16, 18, 6 and 11 was included in public vaccination programs. Considering that Northeast Brazilian women infected with oncogenic NV HPV types as well as genotype covered by 9-valent vaccine have a significantly risk to harbor cervical lesions, new vaccination strategies are needed to avoid HPV type replacement. Recently, the U.S. Food and Drug Administration [69] approved Gardasil 9-Valent vaccine (Human Papillomavirus 9-valent Vaccine, Recombinant), covering five more HPV types than quadrivalent vaccine (HPV types 31, 33, 45, 52 and 58). Gardasil 9 prevent 97% of cervical, vulvar and vaginal cancers and could be a more useful alternative in Northeast Brazilian women, although this vaccine does not protect against HPV56 and HPV51.

Finally we have to recognize the limitations of our study including lack of information on cigarette smoking and alcohol habits, age at first intercourse, marital status, due to the possibility not providing these information in the questionnaire administered to the patients; all these factors are known to possibly influence the risk of HPV infection and cervical lesions/cervical cancer development.

In conclusion, we have demonstrated that women infected with single and multiple VT HPV as well as NV HPV types have a risk to harbour cervical lesions. Longitudinal studies with these HPV types, could clarify the risk of these HPV types to developing cervical lesions in Northeast Brazilian women.

## Supporting Information

S1 File Supporting Information. Table A, HPV diversity as single and multiple infections found in women from the Northeast Brazil. Table B, Distribution of HPV types among normal cytology, High-grade squamous intraepithelial lesion (HSIL) and Low-grade squamous intraepithelial lesion (LSIL), considering single and multiple infections.
(DOCX)
