# Supplementary material for: Association Study between Cervical Lesions and Single or Multiple Vaccine-Target and Non-Vaccine Target Human Papillomavirus (HPV) Types in Women from Northeastern Brazil
Source: PLoS One. 2015 Jul 15;10(7):e0132570. doi: 10.1371/journal.pone.0132570 (PMC4503727; doi:10.1371/journal.pone.0132570)
Supplement: S1 File — Table A, HPV diversity as single and multiple infections found in women from the Northeast Brazil. Table B, Distribution of HPV types among normal cytology, High-grade squamous intraepithelial lesion (HSIL) and Low-grade squamous intraepithelial lesion (LSIL), considering single and multiple infections. (DOCX) [file pone.0132570.s001.docx]

S1 File. HPV diversity as single and multiple infections found in women from the Northeast Brazil (Table A). Distribution of HPV types among normal cytology, High-grade squamous intraepithelial lesion (HSIL) and Low-grade squamous intraepithelial lesion (LSIL), considering single and multiple infections (Table B).

**Table A**

| **HPV types** |  | **Cases** | |
| --- | --- | --- | --- |
|  |  | **n** | **%** |
| *Single infection* (N=158) |  |  |  |
| HPV-6 |  | 7 | 1.9 |
| HPV-11 |  | 2 | 0.5 |
| HPV-16 |  | 28 | 7.6 |
| HPV-18 |  | 6 | 1.6 |
| HPV-31 |  | 48 | 13 |
| HPV-33 |  | 3 | 0.8 |
| HPV-35 |  | 8 | 2.2 |
| HPV-39 |  | 2 | 0.5 |
| HPV-45 |  | 1 | 0.3 |
| HPV-51 |  | 9 | 2.4 |
| HPV-52 |  | 5 | 1.3 |
| HPV-53 |  | 5 | 1.3 |
| HPV-56 |  | 10 | 2.7 |
| HPV-59 |  | 3 | 0.8 |
| HPV-66 |  | 7 | 1.9 |
| HPV-68 |  | 2 | 0.5 |
| HPV-70 |  | 5 | 1.3 |
| HPV-73 |  | 4 | 1.1 |
| HPV-82 |  | 3 | 0.8 |
| *Multiple infections* (N=212) |  |  |  |
| HPV-11/13 |  | 1 | 0.3 |
| HPV-11/16 |  | 3 | 0.8 |
| HPV-11/31 |  | 2 | 0.5 |
| HPV-11/56 |  | 1 | 0.3 |
| HPV-11/35 |  | 1 | 0.3 |
| HPV-11/82 |  | 1 | 0.3 |
| HPV-11/31/58 |  | 1 | 0.3 |
| HPV-11/33/51 |  | 1 | 0.3 |
| HPV-11/45/68 |  | 1 | 0.3 |
| HPV-11/52/53/59/68/73 |  | 1 | 0.3 |
| HPV-16/31 |  | 3 | 0.8 |
| HPV-16/11 |  | 2 | 0.5 |
| HPV-16/18 |  | 1 | 0.3 |
| HPV-16/33 |  | 1 | 0.3 |
| HPV-16/56 |  | 2 | 0.5 |
| HPV-16/58 |  | 3 | 0.8 |
| HPV-16/59 |  | 1 | 0.3 |
| HPV-16/66 |  | 1 | 0.3 |
| HPV-16/18/31 |  | 1 | 0.3 |
| HPV-16/18/39 |  | 1 | 0.3 |
| HPV-16/18/31/58 |  | 1 | 0.3 |
| HPV-16/18/31/35/66/70/82 |  | 1 | 0.3 |
| HPV-16/18/51/53/56 |  | 1 | 0.3 |
| HPV-16/18/53/56 |  | 1 | 0.3 |
| HPV-16/31/35 |  | 2 | 0.5 |
| HPV-16/31/56 |  | 6 | 1.6 |
| HPV-16/31/35/45 |  | 1 | 0.3 |
| HPV-16/31/35/56 |  | 1 | 0.3 |
| HPV-16/31/35/58 |  | 1 | 0.3 |
| HPV-16/31/35/66/67 |  | 1 | 0.3 |
| HPV-16/31/52/56 |  | 2 | 0.5 |
| HPV-16/31/52/73/82/53/66/70 |  | 1 | 0.3 |
| HPV-16/31/56/58 |  | 2 | 0.5 |
| HPV-16/45/51/58 |  | 1 | 0.3 |
| HPV-16/52/82 |  | 1 | 0.3 |
| HPV-16/52/56/68 |  | 1 | 0.3 |
| HPV-16/53/70 |  | 1 | 0.3 |
| HPV-16/56/58 |  | 1 | 0.3 |
| HPV-16/56/58/68 |  | 1 | 0.3 |
| HPV-16/59/56 |  | 1 | 0.3 |
| HPV- 18/31 |  | 5 | 1.3 |
| HPV-18/35 |  | 1 | 0.3 |
| HPV-18/56 |  | 1 | 0.3 |
| HPV-18/59 |  | 2 | 0.5 |
| HPV-18/73 |  | 1 | 0.3 |
| HPV-18/31/35 |  | 2 | 0.5 |
| HPV-18/31/39 |  | 1 | 0.3 |
| HPV-18/31/56 |  | 1 | 0.3 |
| HPV-18/31/59 |  | 1 | 0.3 |
| HPV-18/31/51/52/59 |  | 1 | 0.3 |
| HPV-18/31/52/59 |  | 1 | 0.3 |
| HPV-18/31/56/59 |  | 1 | 0.3 |
| HPV-18/51/73 |  | 1 | 0.3 |
| HPV-18/51/56/66 |  | 1 | 0.3 |
| HPV-18/52/59 |  | 1 | 0.3 |
| HPV-18/52/53/66/68 |  | 1 | 0.3 |
| HPV-18/52/59/68 |  | 1 | 0.3 |
| HPV-18/52/82 |  | 1 | 0.3 |
| HPV-18/56/58/59/73 |  | 1 | 0.3 |
| HPV-26/35 |  | 1 | 0.3 |
| HPV-31/33 |  | 2 | 0.5 |
| HPV-31/35 |  | 1 | 0.3 |
| HPV- 31/51 |  | 2 | 0.5 |
| HPV-31/53 |  | 3 | 0.8 |
| HPV-31/55 |  | 1 | 0.3 |
| HPV-31/56 |  | 9 | 2.4 |
| HPV-31/58 |  | 5 | 1.3 |
| HPV- 31/59 |  | 2 | 0.5 |
| HPV-31/66 |  | 3 | 0.8 |
| HPV-31/70 |  | 2 | 0.5 |
| HPV-31/73 |  | 2 | 0.5 |
| HPV-31/16/18 |  | 1 | 0.3 |
| HPV-31/32/33 |  | 1 | 0.3 |
| HPV-31/33/52 |  | 1 | 0.3 |
| HPV- 31/33/53 |  | 1 | 0.3 |
| HPV-31/33/53/66/82 |  | 1 | 0.3 |
| HPV-31/33/56/66 |  | 1 | 0.3 |
| HPV-31/35/52 |  | 1 | 0.3 |
| HPV-31/35/58 |  | 2 | 0.5 |
| HPV-31/35/52/58 |  | 1 | 0.3 |
| HPV-31/35/56/70 |  | 1 | 0.3 |
| HPV-31/35/58/66 |  | 1 | 0.3 |
| HPV-31/35/58/68 |  | 1 | 0.3 |
| HPV-31/51/58 |  | 1 | 0.3 |
| HPV-31/51/58/59 |  | 1 | 0.3 |
| HPV-31/52/53 |  | 2 | 0.5 |
| HPV-31/53/56/66 |  | 1 | 0.3 |
| HPV-31/56/45 |  | 1 | 0.3 |
| HPV-31/56/52 |  | 1 | 0.3 |
| HPV- 31/56/16/52 |  | 1 | 0.3 |
| HPV-31/56/6/18/53/73 |  | 1 | 0.3 |
| HPV-31/32/33/35/56 |  | 1 | 0.3 |
| HPV-33/35/51/56 |  | 1 | 0.3 |
| HPV-33/53/68 |  | 1 | 0.3 |
| HPV-35/51 |  | 1 | 0.3 |
| HPV-35/68 |  | 2 | 0.5 |
| HPV-35/31/56 |  | 1 | 0.3 |
| HPV-35/52/58 |  | 1 | 0.3 |
| HPV-35/56/58 |  | 1 | 0.3 |
| HPV-39/45 |  | 1 | 0.3 |
| HPV-39/56 |  | 1 | 0.3 |
| HPV-45/51 |  | 1 | 0.3 |
| HPV-45/66 |  | 1 | 0.3 |
| HPV-5/51/82 |  | 1 | 0.3 |
| HPV-51/73 |  | 1 | 0.3 |
| HPV-51/52/58 |  | 2 | 0.5 |
| HPV-51/52/53/58/66 |  | 1 | 0.3 |
| HPV-52/53 |  | 2 | 0.5 |
| HPV-52/56 |  | 1 | 0.3 |
| HPV-52/70 |  | 1 | 0.3 |
| HPV-53/56 |  | 1 | 0.3 |
| HPV-53/56/73 |  | 1 | 0.3 |
| HPV-56/59 |  | 1 | 0.3 |
| HPV-56/66 |  | 1 | 0.3 |
| HPV-56/73 |  | 2 | 0.5 |
| HPV-56/6/16 |  | 1 | 0.3 |
| HPV-56/70/35 |  | 1 | 0.3 |
| HPV-58/56 |  | 1 | 0.3 |
| HPV-58/66 |  | 1 | 0.3 |
| HPV-58/66/68 |  | 2 | 0.5 |
| HPV-58/70/82 |  | 1 | 0.3 |
| HPV-59/70 |  | 1 | 0.3 |
| HPV-59/73 |  | 1 | 0.3 |
| HPV-6/18 |  | 1 | 0.3 |
| HPV-6/31 |  | 3 | 0.8 |
| HPV-6/53 |  | 5 | 1.3 |
| HPV-6/56 |  | 1 | 0.3 |
| HPV-6/59 |  | 1 | 0.3 |
| HPV-6/66 |  | 1 | 0.3 |
| HPV-6/11/53 |  | 1 | 0.3 |
| HPV-6/11/53/56 |  | 1 | 0.3 |
| HPV-6/11/53/70 |  | 1 | 0.3 |
| HPV-6/16/66 |  | 1 | 0.3 |
| HPV-6/16/31/58 |  | 1 | 0.3 |
| HPV-6/16/53 |  | 1 | 0.3 |
| HPV-6/18/31/58 |  | 1 | 0.3 |
| HPV-6/26/51 |  | 2 | 0.5 |
| HPV-6/26/53/70 |  | 1 | 0.3 |
| HPV-6/31/51 |  | 1 | 0.3 |
| HPV-6/31/53 |  | 2 | 0.5 |
| HPV-6/31/58 |  | 1 | 0.3 |
| HPV-6/31/59 |  | 1 | 0.3 |
| HPV-6/31/39/66/68 |  | 1 | 0.3 |
| HPV-6/31/40/56 |  | 1 | 0.3 |
| HPV-6/31/45/58 |  | 1 | 0.3 |
| HPV-6/31/51/52/68 |  | 1 | 0.3 |
| HPV-6/31/58/66 |  | 1 | 0.3 |
| HPV-6/33/51 |  | 1 | 0.3 |
| HPV-6/35/31/53 |  | 1 | 0.3 |
| HPV-6/51/53/59/73 |  | 1 | 0.3 |
| HPV-6/53/68 |  | 1 | 0.3 |
| HPV-6/59/73 |  | 1 | 0.3 |
| HPV-66/73 |  | 1 | 0.3 |
| Total |  | 370 | 100 |

Table B

| **HPV types** |  | **Cases** | |
| --- | --- | --- | --- |
|  |  | **n** | **%** |
| *Normal cytology and Single infection* (N=98) |  |  |  |
| HPV-6 |  | 6 | 6.1 |
| HPV-11 |  | 2 | 2.0 |
| HPV-16 |  | 15 | 15.5 |
| HPV-18 |  | 4 | 4.1 |
| HPV-31 |  | 31 | 31.9 |
| HPV-33 |  | 1 | 1.0 |
| HPV-35 |  | 5 | 5.2 |
| HPV-39 |  | 2 | 2.0 |
| HPV-45 |  | 1 | 1.0 |
| HPV-51 |  | 6 | 6.2 |
| HPV-52 |  | 1 | 1.0 |
| HPV-53 |  | 3 | 3.1 |
| HPV-56 |  | 5 | 5.2 |
| HPV-59 |  | 3 | 3.1 |
| HPV-66 |  | 3 | 3.1 |
| HPV-68 |  | 1 | 1.0 |
| HPV-70 |  | 4 | 4.1 |
| HPV-73 |  | 3 | 3.1 |
| HPV-82 |  | 2 | 2.0 |
| *Normal cytology and Multiple infections* (N=115) |  |  |  |
| HPV-6/53 |  | 4 | 3.5 |
| HPV-31/53 |  | 3 | 2.6 |
| HPV-31/56 |  | 3 | 2.6 |
| Two HPV types |  | 46 | 40 |
| Three HPV types |  | 35 | 30.4 |
| Four HPV types |  | 16 | 13.9 |
| Five HPV types |  | 6 | 5.2 |
| Six HPV types |  | 1 | 0.9 |
| Seven HPV types |  | 1 | 0.9 |
| *HSIL* *and Single infection* (N=37) |  |  |  |
| HPV-16 |  | 9 | 24.3 |
| HPV-18 |  | 1 | 2.7 |
| HPV-31 |  | 7 | 18.9 |
| HPV-33 |  | 1 | 2.7 |
| HPV-35 |  | 3 | 8.1 |
| HPV-51 |  | 3 | 8.1 |
| HPV-52 |  | 3 | 8.1 |
| HPV-53 |  | 1 | 2.7 |
| HPV-56 |  | 3 | 8.1 |
| HPV-66 |  | 4 | 10.8 |
| HPV-70 |  | 1 | 2.7 |
| HPV-82 |  | 1 | 2.7 |
| *HSIL* *and Multiple infection* (N=53) |  |  |  |
| HPV-31/56 |  | 6 | 11.3 |
| Two HPV types |  | 25 | 47.2 |
| Three HPV types |  | 16 | 30.2 |
| Four HPV types |  | 10 | 18.9 |
| Five HPV types |  | 1 | 1.9 |
| Six HPV types |  | 1 | 1.9 |
| *LSIL* *and Single infection* (N=23) |  |  |  |
| HPV-6 |  | 1 | 4.3 |
| HPV-16 |  | 4 | 17.4 |
| HPV-18 |  | 1 | 4.3 |
| HPV-31 |  | 10 | 43.5 |
| HPV-33 |  | 1 | 4.3 |
| HPV-52 |  | 1 | 4.3 |
| HPV-53 |  | 1 | 4.3 |
| HPV-56 |  | 2 | 8.7 |
| HPV-68 |  | 1 | 4.3 |
| HPV-73 |  | 1 | 4.3 |
| *LSIL* *and Multiple infection* (N=43) |  |  |  |
| HPV-31/58 |  | 3 | 7.0 |
| HPV-31/33 |  | 2 | 4.7 |
| Two HPV types |  | 13 | 30.2 |
| Three HPV types |  | 12 | 27.9 |
| Four HPV types |  | 8 | 18.6 |
| Five HPV types |  | 4 | 9.3 |
| Eight HPV types |  | 1 | 2.3 |
